# Constitutional Treatment in Diseases of the Eye

**Published:** 1905-10-07

**Authors:** 


### Hospital Clinics.

### CONSTITUTIONAL TREATMENT IN DISEASES OF THE EYE.

In a course of lectures on Ocular Therapeutics, Mr. Sydney Stephenson [1] alludes to the local evidences of constitutional disorders that may be found in the eyeball and to the necessity in these circumstances of recognising the claim for general treatment. He deals in particular with syphilis and rheumatism. To acquired syphilis may be due iritis, cyclitis, choroiditis, retinitis, etc., while the inherited form of the disease is the usual cause of interstitial keratitis, is often responsible for choroido-retinitis, and sometimes for an oculo-motor paralysis. To meet these conditions mercury by internal medication is usually sufficient, but in obstinate cases or where there is threatened destruction of sight the intramuscular or intravenous methods of injection may be necessary. Mr. Stephenson has had brilliant results in interstitial keratitis and in choroiditis by the intramuscular treatment. On the whole, however, he prefers intravenous injection, in which he uses the following solution: Mercury cyanide 1 part, sodium chloride 0.75, boiled distilled water 100. The injection may be made into any vein that is sufficiently large and accessible, but the median basilic and median cephalic are the most convenient. The procedure is as follows: A fillet is tied rather tightly round the upper arm and the skin is cleansed and sterilised. The vein is then steadied by the operator's thumb, and the needle pushed obliquely through its anterior wall. Penetration is indicated by the entrance of blood into the glass barrel of the syringe, either at once or on slight withdrawal of the piston. Until this condition is fulfilled the injection should not be made, as the needle may have failed to enter the vein, and the introduction of the mercurial solution into the surrounding connective tissue is apt to cause considerable irritation. When the operator is satisfied, as just explained, that the point of the needle has entered he vein, the fillet is removed from the arm, and the contents of the syringe are slowly pressed into the vessel. Then on withdrawal of the needle the skin puncture may be sealed with collodion in the usual manner. In connection with this fashion of administering mercury it should be remembered that, so used, excess of the drug is usually indicated not by soreness of the gums and salivation but by colic, diarrhœa, or other evidences of intestinal disturbance. Another sign of mercurial action is the formation of a red crescent at the free border of the nails.

Rheumatism undoubtedly plays some part in the causation of affections of the eye, though perhaps not so large a one as is commonly attributed to it. Iridocyclitis, in some cases at least, is undoubtedly rheumatic, whilst episcleritis commonly, and conjunctivitis rarely, are "due to chronic articular rheumatism." Paralyses of the ocular muscles are in a more doubtful position. Certainly in many cases, which at the outset appear to be examples of simple or rheumatic ocular palsy, organic nervous disease such as insular sclerosis or tabes dorsalis develops at a later date.

Aspirin is of great value in the treatment of "rheumatic" affections of the eye. In doses of 15 to 30 grains three or four times a day it promptly relieves pain, and without any of the unpleasant symptoms which are apt to attend the use of sodium salicylate.

[1] Medical Press and Circular, August 30, 1905.

